# Artificial neural network to guide intracorneal ring segments implantation for keratoconus treatment: a pilot study

**DOI:** 10.1186/s40662-020-00184-5

**Published:** 2020-04-09

**Authors:** Chiara Fariselli, Alfredo Vega-Estrada, Francisco Arnalich-Montiel, Jorge L. Alio

**Affiliations:** 1 Research and Development Department, Vissum, Calle Cabañal, 1. Edificio Vissum, 03016 Alicante, Spain; 2 Department of Experimental, Diagnostic, and Specialty Medicine (DIMES), Ophthalmology Service, St. Orsola-Malpighi Teaching Hospital, University of Bologna, Bologna, Italy; 3 Cornea and Refractive Surgery Department, Vissum, Alicante, Spain; 4 Division of Ophthalmology, Universidad Miguel Hernández, Alicante, Spain; 5 Cornea and Refractive Surgery Department, Vissum, Madrid, Spain

**Keywords:** Keratoconus, Intracorneal ring segments, Artificial intelligence, Visual acuity, Corneal aberrations

## Abstract

**Background:**

To analyze the clinical results of an artificial neural network (ANN) that has been processed in order to improve the predictability of intracorneal ring segments (ICRS) implantation in keratoconus.

**Methods:**

This retrospective, comparative, nonrandomized, pilot, clinical study included a cohort of 20 keratoconic eyes implanted with intracorneal ring segments KeraRing (Mediphacos, Belo Horizonte, Brazil) using the ANN (ANN group) and 20 keratoconic eyes implanted with KeraRing using the manufacturer’s nomograms (nomogram group). Uncorrected distance visual acuity (UDVA), corrected distance visual acuity (CDVA) (visual acuity is expressed in decimal value and in LogMAR value in brackets), manifest refraction, corneal topography, tomography, aberrometry, pachymetry and volume analysis (Sirius System. CSO, Firenze, Italy) were performed during the preoperative visit; and the two groups, ANN group and nomogram group, did not differ significantly preoperatively in all of the parameters evaluated. These preoperative values were compared with the results obtained at the third-month visit. Mann-Whitney test and Wilcoxon test were used for the statistical analyses.

**Results:**

The spherical equivalent and the keratometric values decreased significantly in both groups. The CDVA improved from 0.60 ± 0.23 (0.22 LogMAR) pre-operatively to 0.73 ± 0.21 (0.14 LogMAR) post-operatively in the ANN group (*p* < 0.005), and from 0.54 ± 0.19 (0.27 LogMAR) pre-operatively to 0.62 ± 0.19 (0.21 LogMAR) post-operatively in the nomogram group (*p* < 0.01), with statistically significant difference between the two groups (*p* < 0.05), being better in the ANN group. Coma-like aberrations decreased significantly in the ANN group, while in the nomogram group they did not change significantly, but no statistically significant difference was found between the two groups.

**Conclusions:**

ANN to guide ICRS provides an increase in the visual acuity, reduction in the spherical equivalent and improvement in the optical quality of keratoconus patients. ANN gives better results when compared with the manufacturer’s nomograms in terms of better corrected vision and reduction of the coma-like aberrations. The constant inclusion of new cases will make the predictability of ANN increasingly better as the software finetunes its learning.

## Background

Keratoconus is an ectatic corneal disorder characterized by a progressive corneal thinning that results in corneal protrusion, irregular astigmatism, and decreased vision [1]. The out-bulging of the cornea into a conical shape is caused by a degradation of the extracellular matrix (ECM), with consequent loss of collagen fibril orientation [2] and biomechanical weakening [3]. As a consequence, the worsening of the ectatic disease is noticed by the patient as a progressive decrease in quality and quantity of vision. For the management of keratoconus, different therapeutic options are available, such as rigid gas-permeable contact lenses, corneal collagen cross-linking (CXL), intracorneal ring segments (ICRS) implantation and keratoplasty. ICRS represents an additive surgical procedure, which improves visual outcome and contact lens tolerance, re-shaping highly distorted corneal surfaces [4] and redistributing the asymmetrical corneal stress caused by the biomechanical decompensation [5]. They have shown safety, reversibility and stability [6, 7], and can delay, and sometimes avoid, corneal grafting in keratoconus patients [8]. ICRS induce a remodeling of anterior and posterior corneal surfaces, which improves the optical quality of the cornea and reduces optical aberrations [9]. The characteristics of ICRS to be implanted, including number, arc length and thickness, are chosen in the majority of cases according to the manufacturer’s nomogram, which most of the time is based on data with poor predictability in keratoconus cases such as refraction and astigmatism, and are empirical, and sometimes based on the experience of the surgeon, which can be subjective.

An artificial neural network (ANN) is a computational program which simulates the decision process in networks of nerve cells of the biological central nervous system [10]. The program receives input and is able to change its internal state (*activation*) according to that input; in response, it produces output depending on the input and activation. The functions that compute the activation can be modified by a process called *learning*. The system, in fact, “learns” to perform tasks by considering examples and automatically generates identifying characteristics from the learning material that it processes [11]. Therefore, the purpose of the present study is to analyze the clinical results of an ANN that has been processed in order to improve the predictability of the results after ICRS implantation. In this case, the artificial intelligence simulates which combinations of segments could provide the best topographic outcome, but also the best corneal optical quality, and consequently the best quality of vision for the patient.

## Methods

### Patients selection

This retrospective, comparative, nonrandomized, pilot, clinical study enrolled a cohort of 20 keratoconic eyes implanted with intracorneal ring segments using the ANN (ANN group), and 20 keratoconic eyes implanted with intracorneal ring segments using the manufacturer’s nomograms (nomogram group) as a control group. Eyes which received only intracorneal rings formed the ICRS group, while eyes which underwent intracorneal rings associated with CXL belonged to the ICRS+CXL group. Before surgery, each patient was informed about the benefits and risks of the surgical procedure. All patients gave informed consent where they agreed that their clinical data may be included in scientific studies. The procedures of the investigation conformed to the tenets of the Declaration of Helsinki and the Ethical Board Committee of Vissum Institution Alicante approved the retrospective revision of the clinical data. Patients were included through a retrospective review of all cases operated with ICRS implantation from January 2017 to May 2019. For all patients the same protocol for data recording and analysis was followed. Only patients with no corneal scars, no previous ocular surgery, and no active ocular disease other than keratoconus were included. The patients wearing contact lenses were instructed in all cases to discontinue their use for at least 2 weeks before each examination for soft contact lenses and at least 4 weeks before each examination for rigid gas-permeable contact lenses. ICRS implantation was indicated because of confirmed keratoconus diagnosis (based on corneal topography and slit-lamp observation), poor motivation of the patient to wear contact lenses, or contact lens intolerance. All ICRS (KeraRing, Mediphacos, Belo Horizonte, Brazil) were implanted using femtosecond laser technology. CXL was indicated when a progression of the disease was detected, according to the keratoconus progression criteria, which included at least 1 diopter (D) increase in simulated keratometry in the steepest meridian and 1 D of astigmatism increase in manifest subjective refraction.

### Examination protocol

Data from the preoperative visit, the first postoperative day and the third postoperative month were recorded in a standardized database. On the first postoperative day, slit-lamp examination was performed in order to verify intracorneal ring position and corneal integrity. During the preoperative visit and the third-postoperative-month visit, all patients underwent a full ocular, refraction and ophthalmoscopic examination, including uncorrected distance visual acuity (UDVA) and corrected distance visual acuity (CDVA) in decimal scale (the corresponding LogMAR value is reported in brackets), manifest refraction (sphere, cylinder and spherical equivalent), slit-lamp biomicroscopy, Goldmann tonometry and fundus evaluation. Patients were classified according to the degree of visual limitation: grade I, patients with spectacle CDVA of 0.90 (0.05 LogMAR) or better; grade II, patients with CDVA equal or better than 0.60 (0.22 LogMAR) and worse than 0.90; grade III, patients with CDVA equal or better than 0.40 (0.4 LogMAR) and worse than 0.60; grade IV, patients with CDVA equal or better than 0.20 (0.7 LogMAR) and worse than 0.40; and grade Plus, patients with CDVA worse than 0.20 [12]. Corneal topography, tomography, aberrometry, pachymetry and volume analysis were performed with the Sirius System (CSO, Firenze, Italy), a topographer which combines a rotating Scheimpflug camera and a Placido disk and allows full analysis of the cornea and anterior segment of the eye. The following topographic variables were evaluated and recorded for analysis:

- *▪ Anterior Corneal Surface keratometry:* simulated mean keratometry in the 3 mm central zone (mean SIM-K), simulated keratometry in the flattest meridian for the 3 mm central zone (K1) and simulated keratometry in the steepest meridian for the 3 mm central zone (K2).
- *▪ Corneal Pachymetry:* central corneal thickness (CCT)
- *▪ Minimal thickness* (ThkMin).
- *▪ Corneal Volume* at a diameter of 10 mm.
- *▪ Corneal Aberrations.* The software of the CSO, the EyeTop2005 (CSO), automatically performs the conversion of corneal elevation profile into corneal wavefront data, using Zernike polynomials with an expansion up to the seventh order. In this study, the root mean square (RMS) values for a 6 mm pupil were calculated for the following types of aberrations: total, higher-order (HOA), astigmatism, coma-like and spherical-like.

### The algorithm

ANNs belongs to the machine learning domain, which is the subfield of artificial intelligence that, as Arthur Samuel said, “gives computers the ability to learn without being explicitly programmed”. In machine learning, the first step is data processing, because the computer must be programmed to optimize a performance using data from past experience; and this data processing is based on the weights of connections between neurons [13]. After the algorithm is prepared and trained, the new data are fed into the network, and the program is able to change its behavior based on what it learns.

In this specific algorithm, the use of the ANN is central, and its aim is to simulate the morphological effect of the implant of one or two of following ring segments: simulated segment types are SI5–90, SI5–120, SI5–160, SI5–210, SI6–90, SI6–120, SI6–150 and SI6–210 where SI5 or SI6 indicates whether the segment has a diameter of 5 or 6 mm followed by its amplitude. Due to the great variability of surgical results after the ICRS implantation, the only way to improve the predictability of the outcomes is to plan the surgery upon experience, and this is the mode of operation of this specific embodiment. Only successful cases were used to feed the brain of the neural network, where “success” was defined as those cases that showed 1 of the following characteristics 6 months after the procedure: (1) an improvement in 1 or more lines of uncorrected or corrected distance visual acuity, (2) a decrease in 2 or more diopters of spherical equivalent, (3) a decrease of at least 1 mm of the RMS corneal higher-order or coma-like aberrations. Thus, the ANN, previously trained with a set of 75 successful cases, was implemented as a Feedforward network, and based on backpropagation algorithm with momentum. The input neurons are the Zernike decomposition of the corneal elevation on an 8 mm diameter, the type of segment, its bisecting line and its thickness; the output are the Zernike decomposition of the corneal elevation after the implant on the same area of the input fitting. As the software allows choosing the center of the implant (between corneal vertex, geometric center and pupil center), the Zernike fitting was performed on the preferred position.

Overall, the algorithm performs as follows: for each possible ring segment (one or two segments), for each segment thickness and for each bisecting line the ANN is asked to predict the outcome of the surgery in terms of elevations; from its output, a corneal wave front is calculated and its point spread function (PSF) is derived. The set of parameters showing the best Strehl ratio is selected as the suggested surgery strategy. As an option, the surgeon can choose whether to consider or ignore the second order, optimizing UDVA or CDVA. Thus, ANN simulates which combinations of segments could provide the best topographic outcome, but also the best corneal optical quality, and consequently the best quality of vision, as a function of the Strehl ratio, for the patient.

### Surgical technique

In all cases, an antibiotic prophylaxis with topical ciprofloxacin was prescribed every 8 h for 2 days before the surgery. All procedures were performed under topical anesthesia. In all cases, the procedure was performed assisted by 60 kHz IntraLase femtosecond system (IntraLase Corp, Irvine, California, USA). After marking the center of the pupil, the disposable suction ring was applied and centered. Then, the disposable glass lens of the laser system was used to applanate the cornea, fixate the eye and help maintain a precise distance from the laser head to the focal point [9]. A continuous circular stromal tunnel was created at approximately 80% of corneal depth, by photodisruption: the laser beam forms a dissection plane, created by the interconnection of microbubbles of carbon dioxide and water vapor. In all eyes, the power used to create the tunnel and the incision was 5 mJ. The procedure lasted approximately 15 s. Five minutes later, the intracorneal ring segments were placed using special forceps and a Sinskey hook. The selection of the number (1 or 2), arc length, and thickness of ICRS was performed following the manufacturer’s nomograms in the nomogram group and following the Artificial Neural Network in the ANN group. Postoperatively, topical tobramycin and dexamethasone eye drops (TobraDex; Alcon Laboratories Inc., Fort Worth, Texas, USA) were used every 6 h for 1 week and then stopped. Topical lubricants were also prescribed every 6 h for 1 month (Systane; Alcon Laboratories Inc).

When the ultraviolet-A/riboflavin mediated corneal collagen CXL was indicated, it was performed immediately after the ICRS implantation, and the “Dresden protocol” was used. After manual abrasion of 8 mm central corneal epithelium, 10 mg riboflavin in 10 mL of 20% dextran solution was applied to the cornea every 2 min for 30 min. Before irradiation, ultrasound pachymetry was performed to ensure a minimum corneal thickness of 400 μm. Then, riboflavin solution was applied every 5 min during the course of a 30-min exposure to 370 nm Ultraviolet A light, with an irradiance of 3 mW/cm^2^. After the treatment, a bandage contact lens was placed until reepithelization.

### Statistical analysis

Statistical analysis was performed using the SPSS statistics software package version 22. Mean values and standard deviations were calculated for every parameter during the follow-up. Nonparametric analyses were performed due to the small sample size. Regarding the comparisons among groups, the nonparametric Mann-Whitney test (Wilcoxon rank-sum test) was applied: nomogram group and ANN group, or ICRS group and ICRS+CXL group (independent samples). Comparisons between pre- and post-operative values within each group were made utilizing the nonparametric Wilcoxon test (paired data). For all statistical tests, the same level of significance was used (*p* < 0.05).

## Results

The current study comprises a cohort of 20 consecutive keratoconic eyes implanted with intracorneal ring segments using the ANN (18 patients; 14 males and 4 females; mean age of 29.6 ± 11.1). Eight eyes of the ANN group received only intracorneal rings (mean age 33.6 ± 12.9) and 12 eyes underwent intracorneal rings associated with CXL (mean age 26.4 ± 8.9). The control group was represented by 20 keratoconic eyes implanted with intracorneal ring segments using the manufacturer’s nomograms (17 patients; 13 males and 4 females; mean age of 35.6 ± 12.4). Fourteen eyes of the nomogram group underwent only ICRS implantation (mean age 34.7 ± 12.5) and 6 eyes, ICRS associated with CXL (mean age 38.8 ± 13.4).

With respect to keratoconus severity, according to the degree of visual limitation, in the ANN group 4 eyes had grade 1 keratoconus, 6 eyes grade 2, 7 eyes grade 3 and 3 eyes grade 4. In the nomogram group, 1 eye had grade 1 keratoconus, 7 eyes had grade 2, 9 eyes grade 3, 2 eyes grade 4 and 1 eye grade Plus.

In the ANN group, considering the two subgroups ICRS group and ICRS+CXL group preoperatively, there were no statistically significant differences in terms of grade of keratoconus, UDVA, CDVA, spherical equivalent, simulated mean keratometry, CCT, ThkMin, corneal volume and corneal aberrometry (Mann-Whitney test, all *p* > 0.05, Table 1). Again, there was no statistically significant difference when we compared the ICRS group and ICRS+CXL group in the postoperative period (Mann-Whitney test, *p* > 0.05, Table 1).

**Table 1:** Comparison between the subgroups of ANN group (ICRS and ICRS+CXL), in preoperative and postoperative period

	**Variables**	**Group**		*p* value
		**ICRS**	**ICRS + CXL**	
**Pre-op**	Grade of keratoconus	2.13 ± 1.13	2.67 ± 0.89	*p* > 0.05
	UDVA [Decimal value (LogMAR)]	0.22 ± 0.14 (0.66)	0.14 ± 0.14 (0.85)	*p* > 0.05
	CDVA [Decimal value (LogMAR)]	0.68 ± 0.24 (0.17)	0.53 ± 0.20 (0.28)	*p* > 0.05
	SE (D)	−2.27 ± 2.92	−4.97 ± 3.82	*p* > 0.05
	Sim-K Avg (D)	46.33 ± 2.79	48.38 ± 4.32	*p* > 0.05
	CCT (μm)	475.63 ± 41.66	473.25 ± 42.28	*p* > 0.05
	ThkMin (μm)	459.63 ± 45.63	456.83 ± 44.48	*p* > 0.05
	Corneal volume (mm^3^)	54.53 ± 3.12	54.14 ± 2.90	*p* > 0.05
	Total aberrations (μm)	4.44 ± 2.03	5.98 ± 2.79	*p* > 0.05
	High Order aberrations (μm)	3.26 ± 1.76	3.98 ± 2.31	*p* > 0.05
	Astigmatism aberrations (μm)	3.38 ± 1.28	3.78 ± 2.93	*p* > 0.05
	Coma-like aberrations (μm)	3.16 ± 1.77	3.84 ± 2.30	*p* > 0.05
	Spherical-like aberrations (μm)	0.81 ± 0.27	1.01 ± 0.38	*p* > 0.05
**Post-op**	UDVA [Decimal value (LogMAR)]	0.37 ± 0.21 (0.43)	0.36 ± 0.22 (0.44)	*p* > 0.05
	CDVA [Decimal value (LogMAR)]	0.78 ± 0.18 (0.11)	0.70 ± 0.24 (0.15)	*p* > 0.05
	SE (D)	−2.39 ± 2.22	−2.71 ± 2.61	*p* > 0.05
	Sim-K Avg (D)	45.07 ± 2.28	45.50 ± 5.37	*p* > 0.05
	CCT (μm)	478.38 ± 37.54	469.25 ± 55.85	*p* > 0.05
	ThkMin (μm)	454.50 ± 47.66	433.75 ± 50.22	*p* > 0.05
	Corneal volume (mm^3^)	55.24 ± 3.72	55.13 ± 3.07	*p* > 0.05
	Total aberrations (μm)	3.91 ± 2.66	5.58 ± 2.72	*p* > 0.05
	High Order aberrations (μm)	2.46 ± 0.73	3.93 ± 2.27	*p* > 0.05
	Astigmatism aberrations (μm)	2.94 ± 2.86	3.81 ± 1.92	*p* > 0.05
	Coma-like aberrations (μm)	2.09 ± 0.51	3.13 ± 1.78	*p* > 0.05
	Spherical-like aberrations (μm)	1.20 ± 0.46	2.22 ± 1.65	*p* > 0.05

In the ANN group, considering the two subgroups ICRS group and ICRS+CXL group, there is no statistically significant difference in terms of grade of keratoconus, UDVA (uncorrected distance visual acuity), CDVA (corrected distance visual acuity), SE (spherical equivalent), Sim-K Avg (simulated mean keratometry), CCT (central corneal thickness), ThkMin (minimal thickness), corneal volume and corneal aberrations, neither in the preoperative nor in the postoperative period (*p* > 0.05). Visual acuity is expressed in decimal value and the corresponding LogMAR value is reported in brackets

The ANN group and the nomogram group did not differ significantly preoperatively in the parameters evaluated: grade of keratoconus, UDVA, CDVA, spherical equivalent, simulated mean keratometry, CCT, ThkMin, corneal volume and corneal aberrometry (Mann-Whitney test, all *p* > 0.05).

No surgical complications occurred during the positioning of the ICRS, and in all cases in which CXL was performed, there was no need for ring repositioning. All eyes showed excellent corneal tolerance with no extrusion or migration of the ring.

### Visual acuity

**Fig. 1:**
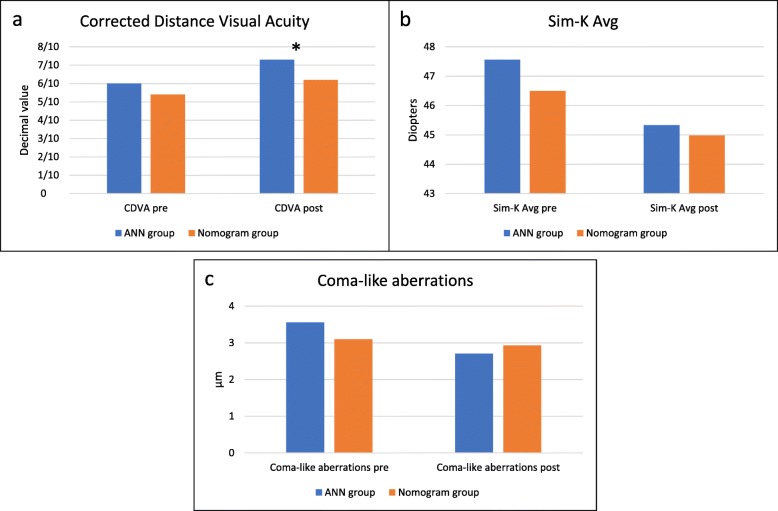
Visual, keratometric and aberrational outcomes after the ICRS implantation. **a** Preoperative and postoperative CDVA in the ANN group and in the nomogram group, with a statistically significant difference (asterisk) in the final visual acuity between the two groups (Mann-Whitney test, *p* < 0.05). **b** Preoperative and postoperative simulated mean keratometry in the 3 mm central zone in the ANN group and in the nomogram group. **c** Preoperative and postoperative coma-like aberrations in the ANN group and in the nomogram group

The average UDVA improved significantly at 3 months postoperatively in the ANN group and in the nomogram group (Wilcoxon test, *p* ≤ 0.001), with no statistically significant difference between the two groups (Mann-Whitney test, *p* > 0.05). The average CDVA improved from 0.60 ± 0.23 (0.22 LogMAR) pre-operatively to 0.73 ± 0.21 (0.14 LogMAR) postoperatively in the ANN group (Wilcoxon test, *p* < 0.005), and from 0.54 ± 0.19 (0.27 LogMAR) pre-operatively to 0.62 ± 0.19 (0.21 LogMAR) postoperatively in the nomogram group (Wilcoxon test, *p* < 0.01), with statistically significant difference between the two groups in term of final visual acuity (Mann-Whitney test, *p* < 0.05), being better in the “ANN group” (Table 2 and Fig. 1a).

**Table 2:** Clinical results of the implantation of ICRS using the ANN and the manufacturer’s nomogram

	**Variables**	**Pre-op**	**Post-op**	*P* value
**ANN group**	UDVA [Decimal value (LogMAR)]	0.17 ± 0.14 (0.77)	0.37 ± 0.21 (0.43)	*p* = 0.001
	CDVA [Decimal value (LogMAR)]	0.60 ± 0.23 (0.22)	0.73 ± 0.21 (0.14)	*p* < 0.005
	SE (D)	−3.89 ± 3.66	−2.58 ± 2.40	*p* < 0.05
	Sim-K1 (D)	45.58 ± 4.05	43.71 ± 4.27	*p* < 0.001
	Sim-K2 (D)	49.80 ± 3.96	47.13 ± 4.56	*p* = 0.001
	Sim-K Avg (D)	47.56 ± 3.84	45.33 ± 4.32	*p* < 0.001
	CCT (μm)	474.20 ± 40.93	472.90 ± 48.44	*p* > 0.05
	ThkMin (μm)	457.95 ± 43.75	442.05 ± 49.05	*p* < 0.01
	Corneal volume (mm^3^)	54.30 ± 2.92	55.17 ± 3.25	*p* < 0.05
	Total aberrations (μm)	5.36 ± 2.57	4.91 ± 2.76	*p* > 0.05
	High Order aberrations (μm)	3.69 ± 2.09	3.34 ± 1.93	*p* > 0.05
	Astigmatism aberrations (μm)	3.62 ± 2.37	3.47 ± 2.31	*p* > 0.05
	Coma-like aberrations (μm)	3.56 ± 2.08	2.71 ± 1.48	*p* < 0.05
	Spherical-like aberrations (μm)	0.93 ± 0.35	1.84 ± 1.41	*p* < 0.005
**Nomogram group**	UDVA [Decimal value (LogMAR)]	0.12 ± 0.11 (0.92)	0.36 ± 0.23 (0.44)	*p* < 0.001
	CDVA [Decimal value (LogMAR)]	0.54 ± 0.19 (0.27)	0.62 ± 0.19 (0.21)	*p* < 0.01
	SE (D)	−4.96 ± 4.16	−1.80 ± 2.48	*p* = 0.001
	Sim-K1 (D)	44.37 ± 4.79	43.15 ± 4.29	*p* > 0.05
	Sim-K2 (D)	48.95 ± 5.48	45.98 ± 4.44	*p* < 0.001
	Sim-K Avg (D)	46.50 ± 4.84	44.98 ± 5.32	*p* < 0.005
	CCT (μm)	457 ± 55.50	453.45 ± 57.25	*p* > 0.05
	ThkMin (μm)	443.55 ± 54.72	431.95 ± 56.43	*p* < 0.05
	Corneal volume (mm^3)^	53.40 ± 3.47	53.46 ± 4.43	*p* > 0.05
	Total aberrations (μm)	5.66 ± 3.54	4.39 ± 2.76	*p* < 0.05
	High Order aberrations (μm)	3.30 ± 2.18	3.42 ± 2.23	*p* > 0.05
	Astigmatism aberrations (μm)	4.16 ± 3.43	2.36 ± 2.18	*p* < 0.005
	Coma-like aberrations (μm)	3.10 ± 2.13	2.93 ± 2.20	*p* > 0.05
	Spherical-like aberrations (μm)	1.05 ± 0.67	1.58 ± 0.89	*p* < 0.005

Preoperatively, the ANN group and the nomogram group do not differ significantly in any of the parameters evaluated (*p* > 0.05). Postoperatively, the ANN group and the nomogram group differed significantly in CDVA (*p* < 0.05). Visual acuity is expressed in decimal value and the corresponding LogMAR value is reported in brackets

### Refraction

The average spherical equivalent decreased significantly by 1.31 D in the ANN group (Wilcoxon test, *p* < 0.05) and by 3.16 D in the nomogram group (Wilcoxon test, *p* = 0.001), with no statistically significant difference between the final spherical equivalent in the two groups (Mann-Whitney test, *p* > 0.05, Table 2).

### Keratometry

The simulated mean keratometry in the 3 mm central zone decreased by 2.23 D in the ANN group (Wilcoxon test, *p* < 0.001) and by 1.52 D in the Nomogram group (Wilcoxon test, *p* < 0.005), with no statistically significant difference between the two groups (Mann-Whitney test, *p* > 0.05) (Table 2 and Fig. 1b).

### Pachymetry and corneal volume

The average central corneal thickness did not change significantly after the ICRS implantation in the two groups, while the minimal thickness decreased significantly. The average corneal volume at a diameter of 10 mm increased in both groups (Table 2).

### Anterior corneal aberrations

In the ANN group, coma-like aberrations decreased significantly, while spherical-like aberrations increased significantly. In the Nomogram group, total aberrations and astigmatism aberrations decreased significantly, spherical-like aberrations increased significantly, while coma-like aberrations did not change significantly after the ICRS implantation. No statistically significant difference was found between the two groups in postoperative aberration (Table 2 and Fig. 1c).

## Discussion

In the present study, we evaluated the outcomes of ICRS implantation in keratoconic eyes made according to the ANN, analyzing if the combination of segments suggested by the artificial intelligence provides an improvement in patient’s vision through a decrease in corneal aberrations.

Some patients received only the ICRS implantation, while others underwent the combination with corneal collagen CXL. Before performing the two different surgical procedures in the ANN group, no statistically significant differences in terms of grade of keratoconus, UDVA, CDVA, spherical equivalent, simulated mean keratometry, CCT, ThkMin, corneal volume and corneal aberrometry were present. Again, comparing the results after the procedures, no statistically significant differences were found. This finding is in accordance with the established absence of a statistical difference in the outcomes between the two treatment strategies, already demonstrated by other authors [14]. As keratoconus is a progressive ectatic disease, reinforcing corneal biomechanical properties with corneal collagen CXL could help to stabilize the corneal ectasia on the long-term [15–17]. While CXL stops or slows the progression of the ectatic process, ICRS implantation flattens and regularizes the cornea and thus, several authors are showing more and more interest in the combination of the two techniques, in order to reap the benefits of both. This has a great relevance, considering that ICRS implants do not ensure control of the ectasia and CXL does not affect visual outcome in patients who underwent the ICRS implantation [18]. Moreover, the combination of intracorneal ring segment implantation and corneal collagen CXL has demonstrated to provide a significant improvement in visual acuity, both UDVA and CDVA, and a significant reduction in spherical equivalent refraction and in keratometry readings, without intraoperative or postoperative complications [19]. These findings suggest that this combination may be an effective and safe treatment for keratoconus correction [19]. Furthermore, the comparison between ICRS implantation only and in combination with corneal collagen CXL has demonstrated similar improvement rates in visual acuity, spherical and cylindrical errors and mean keratometry values [14].

In both groups, the ANN group and the nomogram group, a decrease in keratometric values and spherical equivalent was observed, with an improvement in uncorrected and corrected distance visual acuity. When Colin et al. proposed the use of ICRS for keratoconus treatment, in fact, authors noticed that the flattening in the central cornea, with consequent decrease in keratometric values and astigmatism, led to an improvement in patients’ vision [20]. The remodeling of the topography of anterior and posterior corneal surfaces improves the optical quality of the cornea and reduces optical aberrations, with consequent improvement in CDVA [9]. Several grading systems for keratoconus have been described, according to the different alterations that occur in keratoconic corneas, which induce changes in topographic morphology, corneal keratometry readings and corneal aberrometry [21–23]. But the impairment of the functional performance of the visual system, such as the decrease in visual acuity, caused by the different corneal alterations, is the real cause of visual disability of these patients [12]. Thus, the new grading system, based on visual limitation, has been described, in order to evaluate the disease considering the clinical data, which are more closely related to the disability induced [24]. In addition, the measurement of CDVA, which is objective and readily available in daily practice, can be correlated with other continuous variables, allowing a greater practical use [12]. Our team has recently demonstrated that the evaluation of the results after ICRS implantation should be based on preoperative visual impairment [24], rather than just the geometric assessment of the cornea, which is really unpredictable in keratoconic eyes [20]. In the present study, the eyes implanted according to the ANN reached a significantly better CDVA when compared to the cases operated according to the manufacturer’s nomogram. So, in agreement with the fact that success and failure of ICRS implantation is closely related to the degree of visual limitation [24], and that the decrease in visual acuity is the real cause of the visual disability of keratoconus patients [12], the current results suggest that the combination of segments chosen by the artificial intelligence, providing a greater improvement in patient’s visual acuity, can better reduce the disability induced by keratoconus.

The average central corneal thickness showed no significant decrease after the ICRS implantation in the ANN group and nomogram group, while a significant decrease in minimal corneal thickness was observed, as described by Cakir and colleagues [14]. On the contrary, other authors found that the thinnest pachymetry measures do not change significantly after the treatment [19, 25].

In the ANN group, total anterior corneal aberrations, high order, astigmatism and coma-like aberrations decreased after the ICRS implantation, with, in particular, a statistically significant decrease in coma-like aberrations. In the nomogram group, total aberrations and astigmatism aberrations decreased significantly, while a reduction in the coma-like aberrations was observed although this change was not statistically significant. In both groups, the ICRS implantation induced a statistically significant increase in spherical-like aberrations which can be related to the change in corneal asphericity after the procedure. No statistically significant difference was found between the two groups in the postoperative anterior corneal aberrations.

**Fig. 2:**
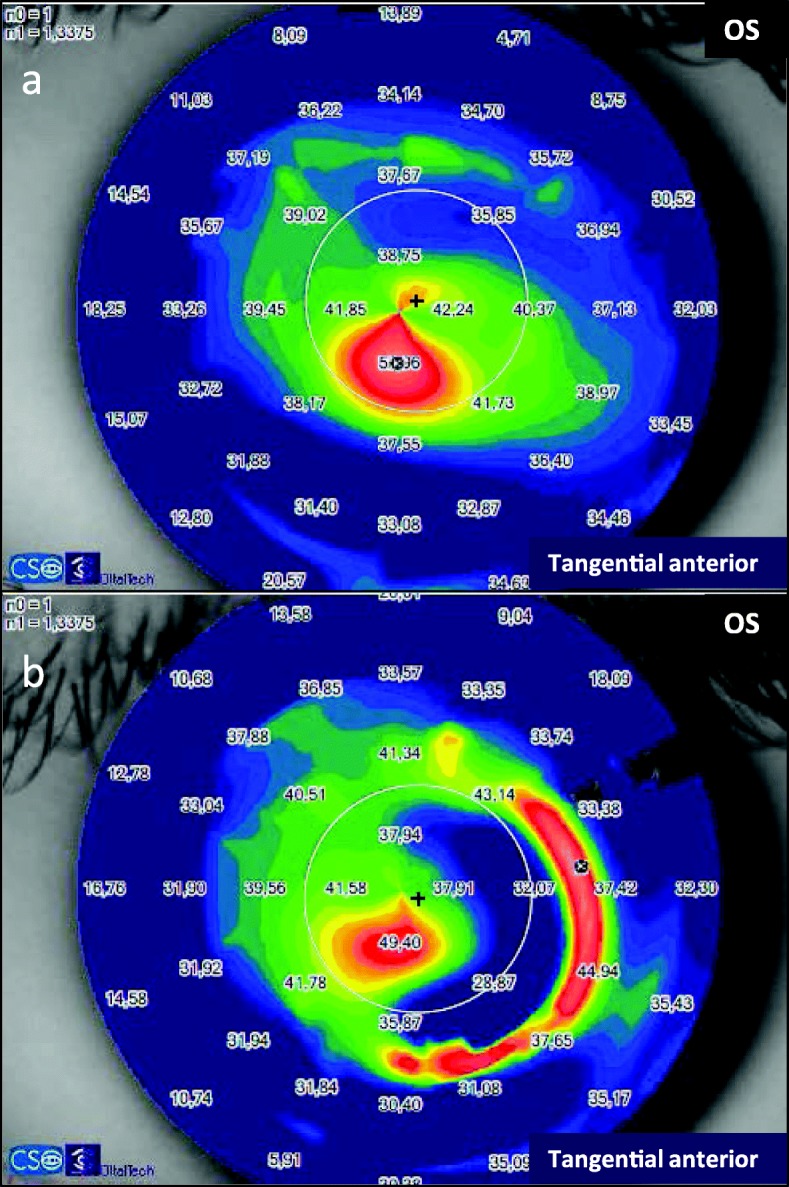
Changes in the tangential anterior map after ICRS implantation according to the ANN’s suggestion. **a** Preoperative tangential anterior map of a keratoconic eye of the ANN group. **b** Postoperative tangential anterior map, after the ICRS implantation following the ANN suggestion

**Fig. 3:**
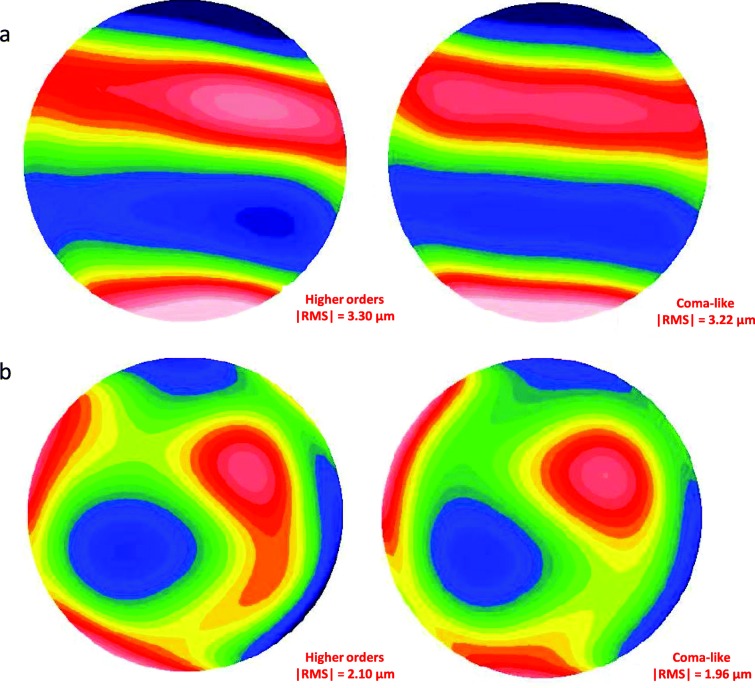
Changes in the aberrometry after ICRS implantation according to the ANN’s suggestion. In the ANN group, total anterior corneal aberrations, high order, astigmatism and coma-like aberrations decreased after the ICRS implantation, with, in particular, a statistically significance in the decrease of coma-like aberrations. **a** Preoperative aberrometry, with a graphic visualization of high order aberrations and coma-like aberrations. **b** Postoperative aberrometry, with a decrease in high order aberrations and coma-like aberrations

**Fig. 4:**
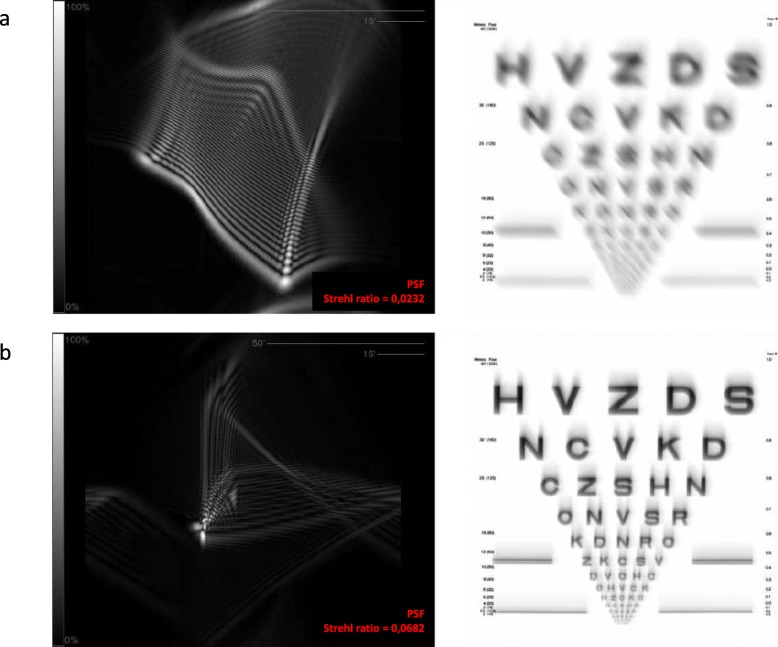
Changes in the quality of retinal image after ICRS implantation according to the ANN’s suggestion. **a** Preoperative Point Spread Function (PSF) and quality of the retinal image. **b** Postoperative PSF and quality of the retinal image, with an increase of the PSF and a significant improvement of the quality of the retinal image

Previous studies have shown a decrease in anterior corneal HOAs, especially the asymmetric aberrations (coma and coma-like), after the regularization of the corneal tissue induced by the ICRS implantation [9, 24, 26]. The patients with the largest decrease in aberrations were those with the most advanced disease [24]. In fact, the higher the preoperative RMS value of the cornea, the more relevant is the reduction in RMS HOA and RMS coma-like aberrations [9]. The significant reduction in total aberrations in the nomogram group is associated with the significant reduction in astigmatism aberrations and could be justified by the fact that the manufacturer’s nomogram is based on refraction and astigmatism. On the other hand, the ANN group presented a significant reduction in coma-like aberrations probably due to the artificial intelligence works suggesting which combination of segments will provide the best quality of vision through a reduction in corneal higher order aberrations. In the current study, the decrease in coma-like aberrations in the ANN group improved the optical quality as a function of the Strehl ratio, and consequently provided a better visual acuity for the keratoconic eye (Figs. 2, 3 and 4). The keratoconic corneas are structurally abnormal and the modeling effect induced by the ICRS implantation is unpredictable [27], with a great variability in the impact on the visual function. But the results of the current study suggest that implanting ICRS according to ANN suggestion can improve the optical quality through a decrease in corneal high order aberrations, with a consequent improvement in visual function and a higher predictability.

Due to the large number of people affected by keratoconus, a great interest is developing around algorithms that could facilitate the diagnosis and also the treatment of the disease. Several machine learning techniques have been proposed as solutions in order to determine whether an eye is affected by keratoconus, using multilayer perceptron, radial basis function network, neural network [28] and support vector machine [29]. For example, the KeratoDetect algorithm is a screening tool based on a learning algorithm that automatically detects the keratoconus disease based on corneal topographies [29]. The use of neural networks was proposed also to determine the keratoconus stage [30] and to predict the evolution of the disease [31]. In particular, Valdés-Mas and colleagues proposed an ANN based on the multilayer perceptron to predict the vision gain of keratoconus patients after ring implantation [31]. This paper presents a neural network approach that does not limit to prediction of vision gain in keratoconus treatment but purposes to improve the outcomes of ICRS implantation. The machine learning ANN suggests which ICRS combination should be implanted to decrease corneal high order aberrations and consequently, to improve optical quality and visual function, increasing also the predictability of the outcomes.

The limitations of the current pilot study are the relatively small size of the sample and the inclusion of both eyes per patient in some cases. However, keratoconus is a bilateral and asymmetric corneal ectatic disease. Therefore, by evaluating both eyes from the same patient is methodologically sound. In the future, when the number of eyes increases, an independent analysis will be performed between one eye per patient and two eyes per patient. Another limitation is the simultaneous presence of patients operated only with the ICRS implantation and other patients who underwent the combination with corneal collagen CXL. Nevertheless, the absence of a statistical difference in the outcomes between the two strategies [14] and the awareness that the best treatment for each patient should always be chosen, explain the recent trend to combine the two methods in the treatment of keratoconus.

The overall results showed a statistically significant improvement of visual outcomes and a reduction in spherical equivalent and keratometry after ICRS implantation in both groups, the one implanted following the manufacturer’s nomogram and the one implanted following ANN. High-order aberrations showed a postoperative reduction only in the ANN group, with statistical significance in coma-like aberrations. The consequent improvement in optical quality could justify the significantly better post-operative CDVA found in the ANN group. Therefore, the artificial intelligence was able to simulate the combination of segments which could provide the best topographic outcome, the best corneal optical quality and consequently, the best vision in patients with keratoconus.

## Conclusions

When the ICRS implantation was guided by the ANN, keratoconic eyes present a significant reduction in coma-like aberrations, with an improvement in the optical quality and consequently in the visual acuity. To plan the surgery upon the results of a large number of previous cases (input) is extremely useful in structurally abnormal corneas such as the keratoconic ones, where the modeling effect induced by the ICRS implantation is unpredictable. Moreover, the activation of the neural network can be enhanced by a learning process because the program is able to change its behavior based on what it learns. Thus, increasing the number of cases used as input by the artificial intelligence makes the output more predictable and accurate, improving the surgical outcome.

## Data Availability

The datasets used and/or analyzed during the current study are available from the corresponding author on reasonable request.

## Abbreviations

ANN: Artificial Neural Network
ICRS: Intracorneal Ring Segments
UDVA: Uncorrected distance visual acuity
CDVA: Corrected distance visual acuity
ECM: Extracellular matrix
CXL: Collagen cross-linking
CCT: Central corneal thickness
ThkMin: Minimal thickness
RMS: Root mean square
HOA: Higher-order aberrations
PSF: Point Spread Function
